# Evaluation of infectious diseases and clinical microbiology specialists’ preferences for hand hygiene: analysis using the multi-attribute utility theory and the analytic hierarchy process methods

**DOI:** 10.1186/s12911-017-0528-z

**Published:** 2017-08-31

**Authors:** Aslı Suner, Ozlem Ege Oruc, Cagri Buke, Hacer Deniz Ozkaya, Gul Kitapcioglu

**Affiliations:** 1 Department of Biostatistics and Medical Informatics, Faculty of Medicine, Ege University, Bornova, İzmir, Turkey; 2 Department of Statistics, Faculty of Science, Dokuz Eylul University, İzmir, Turkey; 3 Department of Infectious Diseases and Clinical Microbiology, Faculty of Medicine, Ege University, İzmir, Turkey; 4 Current address: Department of Infectious Diseases, Yeditepe University Hospital, Yeditepe University, İstanbul, Turkey; 5 Department of Infectious Diseases, Cigli Regional Education Hospital, İzmir, Turkey

**Keywords:** Healthcare-associated infections, Hand hygiene, Analytic hierarchy process, Multi-attribute utility theory

## Abstract

**Background:**

Hand hygiene is one of the most effective attempts to control nosocomial infections, and it is an important measure to avoid the transmission of pathogens. However, the compliance of healthcare workers (HCWs) with hand washing is still poor worldwide. Herein, we aimed to determine the best hand hygiene preference of the infectious diseases and clinical microbiology (IDCM) specialists to prevent transmission of microorganisms from one patient to another.

**Methods:**

Expert opinions regarding the criteria that influence the best hand hygiene preference were collected through a questionnaire via face-to-face interviews. Afterwards, these opinions were examined with two widely used multi-criteria decision analysis (MCDA) methods, the Multi-Attribute Utility Theory (MAUT) and the Analytic Hierarchy Process (AHP).

**Results:**

A total of 15 IDCM specialist opinions were collected from diverse private and public hospitals located in İzmir, Turkey. The mean age of the participants was 49.73 ± 8.46, and the mean experience year of the participants in their fields was 17.67 ± 11.98. The findings that we obtained through two distinct decision making methods, the MAUT and the AHP, suggest that alcohol-based antiseptic solution (ABAS) has the highest utility (0.86) and priority (0.69) among the experts’ choices.

**Conclusion:**

In conclusion, the MAUT and the AHP, decision models developed here indicate that rubbing the hands with ABAS is the most favorable choice for IDCM specialists to prevent nosocomial infection.

**Electronic supplementary material:**

The online version of this article (10.1186/s12911-017-0528-z) contains supplementary material, which is available to authorized users.

## Background

Hand hygiene is one of the most effective attempts to control nosocomial infections, and is performed by washing hands with antimicrobial soap and water, and/or by rubbing with alcohol-based antiseptic solutions (ABAS) [1, 2]. Several publications have appeared in recent years documenting the importance of hand hygiene to prevent and to control the spread of healthcare-associated infections, and there has been a growing interest in this topic [1–3].

The first evidence for the benefits of hand hygiene, implemented by Semmelweis in 1847, demonstrated that cleansing contaminated hands with antiseptic agents was more effective than hand washing with soap and water to reduce healthcare-associated transmission of microorganisms. Today, it is widely accepted that washing hands with antimicrobial soap and water in cases where hands are visibly contaminated with proteinaceous material, including patients’ blood or other body fluids is the best practice. In cases where the hands are not visibly soiled, the use of alcohol based hand rubs for routine hand hygiene in clinical situations is an effective and preferred choice [4, 5]. The World Health Organization (WHO) recommends that hand hygiene should be performed basically in five situations, including (i) before contact with patients, (ii) immediately before aseptic procedures, (iii) immediately after contact with patient’s body fluids, (iv) after contact with patients, and (v) after touching any object or furniture in the patient’s surroundings [6]. Despite the compliance level of healthcare workers (HCWs) should be 100% in all five moments, described by the WHO, it is still poor worldwide and thought that the negative attitudes and lack of motivation of HCWs, and increased workload are the major contributors of low compliance [7]. A comprehensive review on hand hygiene studies by the WHO indicates that the average baseline compliance level of HCWs is 38.7% (ranging from 5 to 89%) [6]. Additionally, in a recent systematic review by Luangasanatip et al. [3], this level can be as low as 34% among HCWs.

Soaps are detergent-based products, and although plain soaps have cleaning activity, they lack the efficacy to remove many hazardous pathogens from the hands of HCWs [8]. On the other hand, alcohols denature proteins, and alcohol solutions containing 60–95% alcohol are the most effective against gram-positive and gram-negative vegetative bacteria, including multidrug-resistant pathogens (e.g., MRSA and VRE), *mycobacterium tuberculosis* and various fungi. As reported by Boyce and Pittet [5] alcohol solutions effectively reduces bacterial count on the hand within 30 s of application. Therefore, hand rubbing with ABAS is the preferred hand hygiene procedure. Its main advantages over soap and water include (i) a higher level of antimicrobial efficacy, (ii) faster usage time, and (iii) easier availability at the point of care [6, 9].

In recent years, multi-criteria decision analysis (MCDA) methods, including the Multi-Attribute Utility Theory (MAUT) and the Analytic Hierarchy Process (AHP), have gained popularity in a wide range of fields of healthcare, in which a number of criteria must be taken into account while making crucial decisions. Previous reports indicate that the MAUT method has been successfully applied to solving numerous healthcare associated problems, such as formulary management in a health system, planning of emergency medical services, decision making in delivery of epidural analgesia during labor and in flu vaccination, and the treatment of streptococcal sore throat, rheumatic fever, schizophrenia and cancer [10–18]. Another MCDA technique, the AHP method, on the other hand, has been utilized in distinct applications of healthcare, including diagnosis, treatment, priority setting, healthcare management and health technology evaluation [19–25].

Given the substantial contribution of hand hygiene, a simple and low-cost action, to preventing and controlling the spread of healthcare-associated infections, the evaluation of the choice of hand hygiene agents among the infectious diseases and clinical microbiology (IDCM) specialists is crucial. So far, a number of studies have investigated the criteria that influence the hand hygiene preference of the IDCM specialists [7, 26–28]. To date, however, there is no available study exploring the priorities among these criteria and the most preferred hand hygiene alternative with MCDA approaches yet. Herein, we evaluated for the first time the best hand hygiene preference of the IDCM specialists with commonly used MCDA techniques, the MAUT and the AHP. To that end, we collected expert opinions via face-to-face interviews, and then modeled these opinions with MCDA methods. We observed that rubbing the hands with ABAS had the highest total utility value, and was the alternative contributing the most to the goal of choosing the best hand hygiene method of the IDCM specialists. The detailed theoretical background of the MAUT and the AHP methods are given in the methods section.

## Methods

**Fig. 1:**
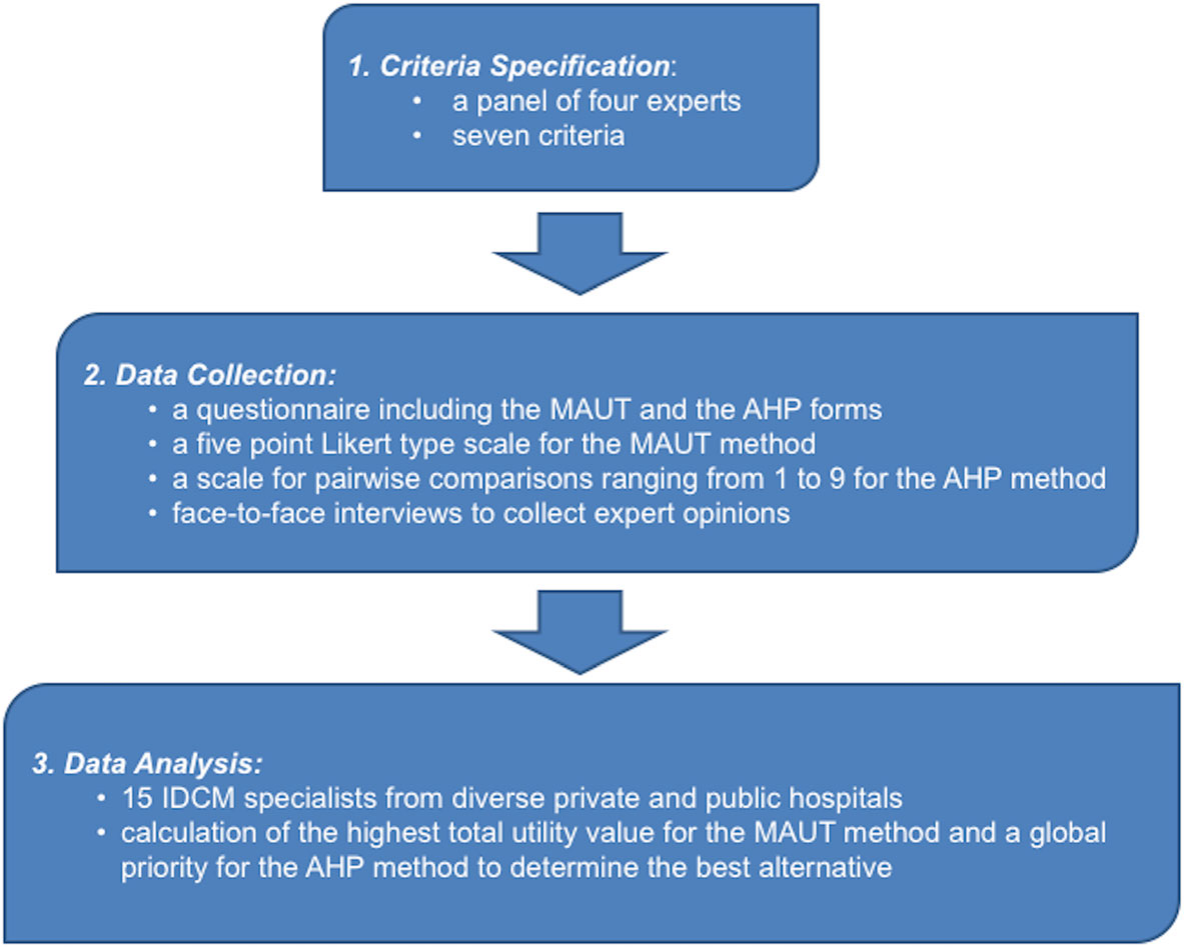
Flow diagram describing study

The study was conducted in three phases, as shown in Fig. 1, to decide the best hand hygiene preference of the experts. The methodology followed in our research includes: (i) criteria specification, (ii) data collection, and (iii) data analysis steps. Each of these steps is explained sequentially in detail below:

### Criteria specification

The criteria that influence the best hand hygiene preference of the IDCM specialists were established by a panel of four experts, who are co-authors of this paper, from different backgrounds, including infections diseases, public health, biostatistics, and statistics. The panel of experts conducted a literature search using PubMed, PubMed Central, and Medline databases, and determined the following seven criteria:

(i) ᅟ**Short time application:** Washing the hands with antimicrobial soap and water must take at least 40 to 60 s rubbing, while ABAS must take at least 20 to 40 s [26].
(ii) ᅟ**Glove usage:** Providing hand hygiene method before and after glove usage [9, 29].
(iii) ᅟ**Dry and cracked skin:** Possible side-effect of washing with soap and water and/or alcohol-based hand antiseptics due to frequent daily usage [30].
(iv) ᅟ**Workload of the health workers:** For all HCWs such as nurses and allied healthcare personnel [27, 28, 30].
(v) ᅟ**Easy to use:** Easy access to materials, inability to easily reach the sink, no suitable water taps for hand hygiene [27, 28, 30].
(vi) ᅟ**Intervention type:** Physical contact with patient (handshake, fever measurement, pulse measurement, blood pressure measurement, carrying patient, etc.), physical contact with surrounding environment of the patient (patient beds, linens, tables, chairs, cabinets, etc.), and between procedures in the same patient (urinary catheter, opening intravenous, inserting a nasogastric tube etc.) [28].
(vii) ᅟ**Efficiency:** More effective than other [26].

### Data collection

A total of 15 IDCM specialists, from diverse public and private hospitals located in İzmir, Turkey, were participated to the study. We collected the expert opinions via face-to-face interviews through a questionnaire (Additional file 1), and these opinions were utilized to develop the hierarchical structure of criteria for the MAUT and the AHP models. The initial part of the questionnaire was regarding the demographic characteristics of experts. The second and the third parts were consisting of a five-point Likert-type scale, ranging from “very important” to “not important at all” for the MAUT method, and a scale for pairwise comparisons, varying from 1 to 9 for the AHP method, respectively. The Cronbach’s alpha (α) values of the items in questionnaire were calculated to test the validity of the MAUT and the AHP forms. Because it was an interview study with experts, no patients were involved in the study, and the expert opinions were analyzed with Microsoft Excel and Expert Choice software for the MAUT method, and the AHP method, respectively. The expert opinions and analysis results for both methods will be made available upon request from the corresponding author.

### Data analysis

#### MAUT method

The MAUT, one of the commonly used MCDA methods, is based on expected utility theory. The method assigns a utility to every possible consequence to decide the best action in a given problem, and then calculates the best possible utility [31, 32]. The MAUT method incorporates the preferences of each consequence at every step and the levels of significance of the criteria are obtained using the entropy metric.

Entropy value calculation has four basic steps, which begins with the construction of a decision matrix and followed by the calculation of normalization values (*r*_*ij*_) for each of the alternatives. Then, the entropy values (*e*_*j*_) of the alternatives for each criterion are determined. In the last step, the weight values (*w*_*j*_) are achieved. The details of calculation steps for entropy are as follows:

*Step 1:* A decision matrix is obtained which contains *m* alternatives and *n* criteria in eq. (1),

1 $$ D=\left[\begin{array}{cccc}\hfill {x}_{11}\hfill & \hfill {x}_{12}\hfill & \hfill \cdots \hfill & \hfill {x}_{1n}\hfill \\ {}\hfill {x}_{21}\hfill & \hfill {x}_{22}\hfill & \hfill \vdots \hfill & \hfill {x}_{2n}\hfill \\ {}\hfill \vdots \hfill & \hfill \vdots \hfill & \hfill \vdots \hfill & \hfill \vdots \hfill \\ {}\hfill {x}_{i1}\hfill & \hfill {x}_{i2}\hfill & \hfill \cdots \hfill & \hfill {x}_{in}\hfill \\ {}\hfill \vdots \hfill & \hfill \vdots \hfill & \hfill \vdots \hfill & \hfill \vdots \hfill \\ {}\hfill {x}_{m1}\hfill & \hfill {x}_{m2}\hfill & \hfill \vdots \hfill & \hfill {x}_{mn}\hfill \end{array}\right] $$

where *x*_*ij*_ is the success value of *i.* alternative for *j.* criteria, *i* = 1,2,...,*m* ve *j* = 1,2,...,*n*.

*Step 2:* Using the following formula in eq. (2), normalized decision matrix values (*R* =[*r*_*ij*_]_*m* × *n*_) are obtained.

2 $$ {r}_{ij}=\frac{x_{ij}}{\sum_{p=1}^m{x}_{pj}},i=1,2,\dots, m\;\mathrm{and}\;j=1,2,\dots, n. $$

*Step 3:* The entropy value for each criterion in eq. (3) are calculated,

3 $$ {e}_j=-\frac{1}{\ln m}{\sum}_{i=1}^m{r}_{ij}\ln {r}_{ij},j=1,2,\dots, n. $$

where *e*_*j*_ is the entropy value of criteria.

*Step 4:* The weights of criteria in eq. (4) are calculated,

4 $$ {W}_j=\frac{1-{e}_j}{\sum_{p=1}^n\left(1-{e}_p\right)},j=1,2,\dots, n. $$

where *W*_*j*_ is the weight value of each criterion and satisfies the following condition in eq. (5).

5 $$ {\sum}_{j=1}^n{W}_j=1 $$

The MAUT method includes three steps: (i) the utility values (*U*_*ij*_) are calculated with the success value of *i.* alternative for *j.* criteria in the decision matrix (ii) the total utility values are obtained in the second step, and (iii) the alternative, which has the highest total utilty value, is determined as the best.

The following equations represent step-by-step calculation of the MAUT method:

*Step 1:* Utility values are determined and *u*_*ij*_ is calculated using eq. (6).

6 $$ {u}_{ij}=\frac{x_{ij}-{x}_{j^{-}}}{x_{{{}_j}^{+}}-{x}_{j^{-}}} $$

where $$ {x}_{{{}_j}^{+}}={\mathrm{max}}_i{x}_{ij} $$ and $$ {x}_{j^{-}}={\mathrm{min}}_i{x}_{ij} $$.

Since we have two alternatives to assess; maximum and minimum utility values are determined as 1 and 0, respectively. If the number of alternatives is more than two, the eq. (6) is used to calculate the utility value.

*Step 2:* Total utility values are obtained as follows:

7 $$ {U}_i={\sum}_{j=1}^n{w}_j{u}_{ij},i=1,2,\dots, m. $$

*Step 3:* Preference ranking is calculated, and the alternative, which results in the highest total utility value, is determined as the best alternative.

#### The AHP method

To make selection decisions from alternatives with pairwise comparisons; the AHP method is an efficient MCDA method for those who study on complex problems. The AHP method, introduced by Thomas Saaty in 1970s, takes decision makers’ subjective and objective knowledge into account, and also checks the consistency of their judgments [33]. The following steps explain the details of the AHP methodology:

*Step 1:* The decision making process of the AHP method starts with problem definition. Then, the hierarchical structure of the model is defined with the following hierarchy (from top to bottom): (i) goal of the problem, (ii) criteria, and (iii) alternatives. After construction of the hierarchy, data analysis of priority determination and consistency confirmation are completed [34]. The priority determination comprises both relative weights of criteria and relative priorities of alternatives.

*Step 2:* The fundamental element of the AHP method is pairwise comparison. The expert judgments are made by using a scale, ranging from 1 to 9 (Table 1). In total, *n* (*n* − 1)/2 judgments are made to create a set of matrices, where *n* is the amount of criteria or alternatives. The scale helps the decision maker to judge how many times more important one criterion or alternative is compared to others. The matrix elements are scale values and indicate the importance of criterion. The matrix of pairwise comparisons also named priority matrix (*n*x*n*) consisting of elements *a*_*ij*_, the importance of criterion *i* to criterion *j* as shown in eq. (8).

8 $$ A=\left[\begin{array}{ccc}\hfill 1\hfill & \hfill \cdots \hfill & \hfill {a}_{1n}\hfill \\ {}\hfill \vdots \hfill & \hfill \ddots \hfill & \hfill \vdots \hfill \\ {}\hfill {a}_{n1}\hfill & \hfill \cdots \hfill & \hfill 1\hfill \end{array}\right] $$

**Table 1:** The scale of the AHP for pairwise comparisons

Importance on a scale	Definition
1	Equal importance
3	Moderate importance
5	Strong importance
7	Very strong or demonstrated importance
9	Extreme importance
2, 4, 6, 8	Intermediate values between the two judgments

The *a*_*ji*_ equals 1/*a*_*ij*_, if a criterion X is *a*_*ij*_ times more important than criterion Y. In this case, the criterion Y must be absolutely 1/*a*_*ij*_ times less important than criterion X. The more theoretical background of the AHP methodology can be reviewed in [33, 35–37].

*Step 3:* A consistency index (CI) is used to evaluate the matrix consistency and calculated as CI = (λ_max_–*n*)/(*n*–1). In this equation, the eigenvalue (λ_max_) is used to assess the consistency of comparisons. A CI is calculated with the eigenvalue, and a random index (RI) is associated with the order n of the matrix (Table 2). Here, *n* (the matrix size) is equal to the number of criteria. The expert judgment’s consistency is measured by consistency ratio (CR) = CI/RI.

**Table 2:** Random Index (RI)

Matrix size (n)	1	2	3	4	5	6	7	8	9	10
Random consistency	0.00	0.00	0.58	0.90	1.12	1.24	1.32	1.41	1.45	1.49

As previously discussed in [24], the weights of each participant’s answers may be equal or not because of their positions in the study, or answers may be achieved as a group decision. In some cases, experts may not accept to make group decisions and prefer to give their individual opinion. In this situation, the results of the pairwise comparisons of each participant can be combined with geometric mean [34]. In our study, the evaluations of the experts’ pairwise comparisons were performed by geometric mean, and analyzed each participant’s answers with an equal weight. Then, the CR was calculated. If the acceptable CR was smaller than 0.10, it was considered as the comparisons of the experts were consistent [34].

*Step 4:* The priorities of each criterion over others and importance of one alternative over another with respect to a common criterion are achieved [38].

## Results

Eleven of the participants (73.3%) were woman and the majority of them (73.3%) were employed in public hospitals. The mean age of the participants was 49.73 ± 8.46, and the mean year of experience was 17.67 ± 11.98. Table 3 shows the demographic characteristics of the participants. The Cronbach’s alpha (α) values of the items in questionnaire were 0.74 and 0.77 for the MAUT and the AHP methods, respectively. These results suggested that the presence of the acceptable internal reliability in the questionnaire [39].

**Table 3:** The demographic characteristics of the participants

Characteristics	Category	Frequency (%)
Gender	Female	11 (73.3%)
	Male	4 (26.7%)
Institution	Public Hospital	11 (73.3%)
	Private Hospital	3 (20.0%)
	Not Specified	1 (6.7%)
Age	Under 50	8 (53.3%)
	50 and more	7 (46.7%)
Experience year	Under 20	8 (53.3%)
	20 and more	7 (46.7%)

### Results of the MAUT method

Table 4 summarizes the results of the MAUT method. The decision matrix was establihed for two alternatives and seven criteria. We calculated the normalization values (*r*_*ij*_) for alternatives using the eq. (2). Then, the entropy values (*e*_*j*_) of the alternatives for each criterion were determined with the eq. (3) and weight values (*w*_*j*_) were obtained using these values. Utility values (*U*_*ij*_) were obtained by using the weight values by the eq. (7) for the criteria. Finally, an alternative, which has the best utiliy, was found.

**Table 4:** Normalized decision matrix values, entropy values and weights of the criteria

Criteria	Norm *r*_*ij*_		Entropy	Weights (w_i_)
	Antimicrobial soap and water	Alcohol-based antiseptic solution (ABAS)		
Short time application	0.43	0.57	0.98	0.22
Glove usage	0.52	0.48	0.99	0.05
Dry and cracked skin	0.55	0.45	0.99	0.09
Workload of the staff	0.43	0.57	0.98	0.22
Easy to use	0.41	0.60	0.97	0.35
Intervention type	0.47	0.53	0.99	0.04
Efficiency	0.47	0.53	0.99	0.03

**Fig. 2:**
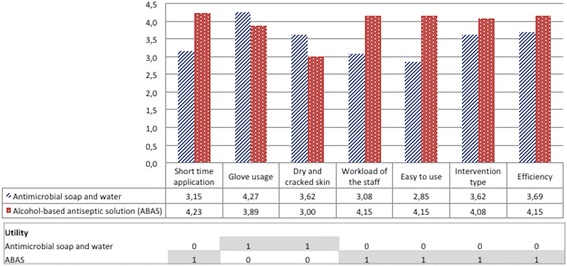
The best and the worst values used in the MAUT method

We found that “glove usage” and “easy to use” criteria had the highest (4.27) and the lowest (2.85) mean values, respectively for antimicrobial soap and water (Fig. 2). However, for ABAS, the MAUT calculation revealed that “short time application” had the highest mean value (4.23) and “dry and cracked skin” had the lowest one (3.00) (Fig. 2). In addition to these, ABAS had the best values in 5 out of 7 criteria. When we calculated the total utility values of both hand hygiene methods with eq. (9) and (10), it was found that rubbing hands with ABAS had the highest total utility value of 0.86.

9 $$ \mathrm{Total}\  \mathrm{utility}\  \mathrm{value}\ \mathrm{for}\ \mathrm{antimicrobial}\  \mathrm{soap}\ \mathrm{and}\ \mathrm{water}=\left(1\times 0.05\right)+\left(1\times 0.09\right)=0.14 $$

10 $$ \mathrm{Total}\  \mathrm{utility}\  \mathrm{value}\ \mathrm{for}\ \mathrm{ABAS}=\left(1\times 0.22\right)+\left(1\times 0.22\right)+\left(1\times 0.35\right)+\left(1\times 0.04\right)+\left(1\times 0.03\right)=0.86 $$

### Results of the AHP method

**Fig. 3:**
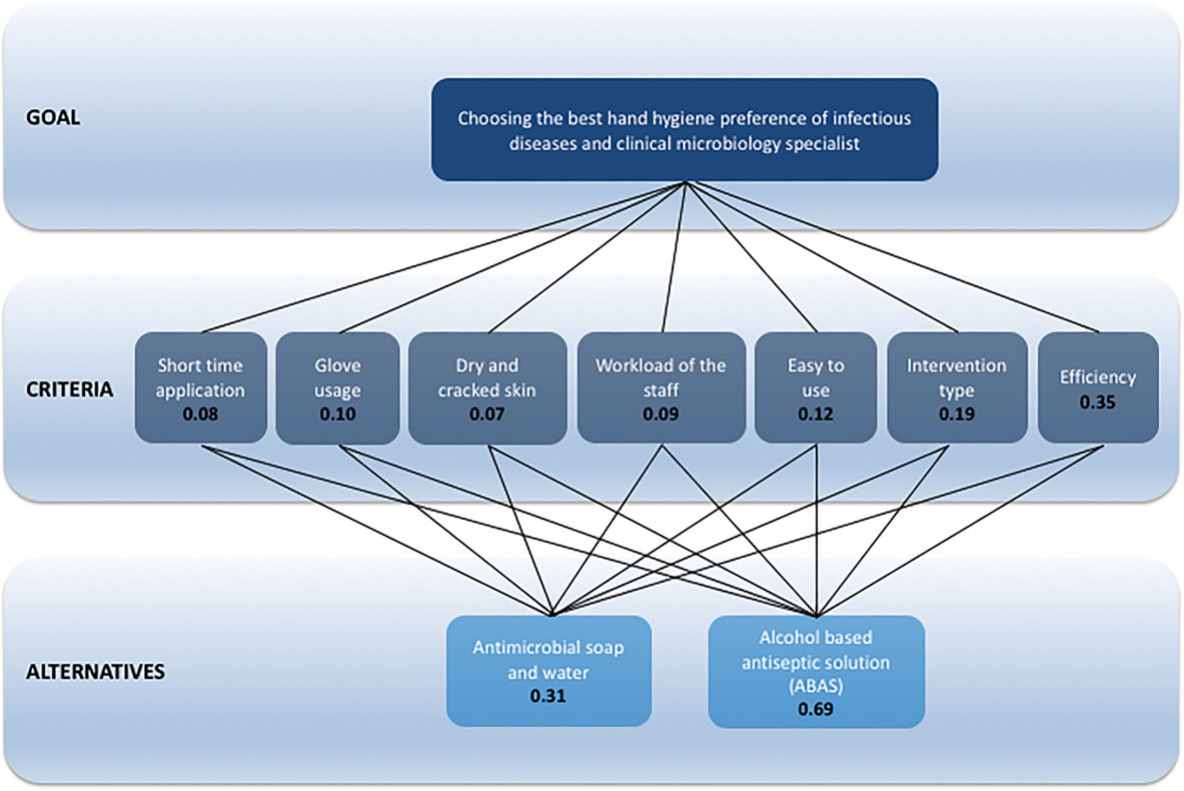
The goal, criteria and alternatives of the AHP model

**Fig. 4:**
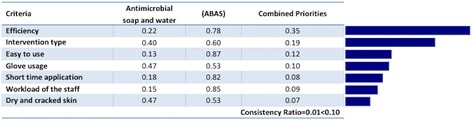
Combined priorities of alternatives according to each criterion for the AHP method

The three levels of the AHP model employed for distinguishing the most favorable hand hygiene method for the IDCM specialists were created as shown in Fig. 3. The participants performed 21 pairwise comparisons between two distinct criteria, and the prioritization tables for each participant were determined. By using CI and RI values for each of seven criteria, the CRs for the AHP model were calculated for each participant’s decisions as shown in Table 5. Then, the paired comparisons matrix of the combined decisions for participants was obtained (Table 6). In Table 6, each number represents the importance of one criterion to other. For example, the IDMC specialists indicated that the “efficiency” criterion was four times more important than “short time application”, “work load of the stuff” and “easy to use” criteria however it was five times less important than “dry and cracked skin” criterion. Additionally, the CR value for the combined priorities of the AHP matrix was determined as 0.01. We therefore considered the combined judgments of the participants to be consistent (Fig. 4).

**Table 5:** CRs for the AHP model for 15 participants

Participant	CR	Participant	CR
1	0.09	9	0.08
2	0.08	10	0.07
3	0.09	11	0.09
4	0.07	12	0.08
5	0.09	13	0.09
6	0.07	14	0.07
7	0.08	15	0.08
8	0.08

**Table 6:** The paired comparisons matrix of the combined decisions for participants

	Short time application	Glove usage	Dry and cracked skin	Workload of the staff	Easy to use	Intervention type	Efficiency
Short time application	1	1	1	1	2	1/2	1/4
Glove usage	1	1	1	1	1	2	3
Dry and cracked skin	1	1	1	1	2	2	5
Workload of the staff	1	1	1	1	1/2	1/2	1/4
Easy to use	1/2	1	1/2	2	1	1/2	1/4
Intervention type	2	1/2	1/2	2	2	1	1/2
Efficiency	4	1/3	1/5	4	4	2	1

**Fig. 5:**
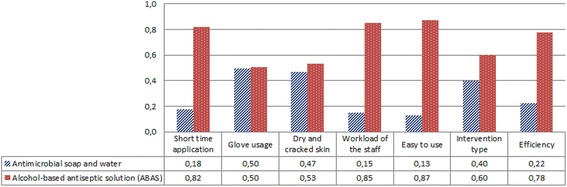
Priorities of alternatives for each criterion for the AHP method

According to the expert preferences, the “efficiency” criterion had the highest relative weight of 0.35, followed by the “intervention type” and “easy to use” criteria, with respective weights of 0.19 and 0.12. On the other hand, the “dry and cracked skin” criterion had the lowest relative weight of 0.07. The ABAS, with a global priority of 0.69, was the alternative contributing the most to the goal of choosing the best hand hygiene method of the IDCM specialists (Fig. 3). The other alternative, antimicrobial soap and water had considerably less priority (0.31). In each case, the best choice for each criterion was the ABAS, and Fig. 5 shows the rankings of the alternatives against the seven covering criteria.

## Discussion

In this study, we utilized the MAUT and the AHP methods to investigate the factors affecting hand hygiene preference of the IDCM specialists, and determined their best choice for the most favorable way to prevent nosocomial infection among HCWs. Hand hygiene in healthcare settings is commonly implemented in two ways; disinfecting hands with an antiseptic agents and washing hands with soap and water. Previous studies comparing hand hygiene by hand rubbing with an ABAS and hand washing with antiseptic soap clearly proved that hand hygiene with hand rubbing procedure was the most appropriate way to reduce the bacterial contamination and to increase hand hygiene compliance [27, 40–42]. In a randomized controlled trial during daily nursing sessions, it was presented that reduction in bacterial contamination was significantly higher with hand rubbing than hand washing (83% vs 58%; *p* = 0.0012), and hand rubbing with an ABAS was preferred to hand washing due to its rapid action and accessibility [26]. Therefore, it has been regarded that hand hygiene by rubbing with ABAS is faster and more effective than that of by washing hands with soap and water [6]. The concurrent evaluation of the decision models established in our study suggests, consistent with previous findings, that the best choice of the IDCM specialists for hand hygiene is ABAS with the highest priority and utility. Our study has also revealed that “efficiency” is the most important factor influencing the preference of hand hygiene. This finding agrees with the results offered by Girou et al. in [26], suggesting that HCWs usually prefer the most effective method.

HCWs have a tendency to overestimate their own compliance [28] and their hand washing habits differ in the five moments of hand hygiene [7, 30, 41]. For example, HCWs potentially prefer to protect themselves rather than patients therefore the reported hand hygiene rates are higher for after patient contact than that of before [7, 28]. In our pairwise comparisons of the AHP method, we found that “efficiency” was less important than “dry and cracked skin” criterion in terms of the hand hygiene alternatives. However, when our criteria list being ranked by their priorities, “efficiency” and “dry and cracked skin” were the most and the least important criteria, respectively. These findings were not surprising, and consistent with earlier researches [7, 28] that HCWs more likely to tend to give priority to protecting themselves and their health. On the other hand, the implication of “efficiency” by IDCMs may be interpreted as a real belief and the evidence of their compliance to hand hygiene to protect their patients since it has long been known that the rubbing the hands with ABAS is more efficient than washing the hands with soap and water. However, this reality does not replace with another truth. Because the ABAS without glycerin, as a humectant, can be harmful for the hands and cause dry and cracked skin. This might be one of the reasons why the effectiveness of hand hygiene is less important when considered the efficiency and the harmful effect of ABAS together.

Although the MCDA methods do not have a clear superiority to each other, the two most commonly used techniques, the MAUT and the AHP, have been particularly preferred in this study [19, 43]. Given the strengths of the AHP, unlike to other approaches, it is a powerful tool in terms of (i) evaluating the priorities of expert opinions with pairwise comparisons, (ii) demonstrating uncertain and conflicting opinions as numerical values, (iii) combining decisions among experts from different or similar expertise, and (iv) having objectivity and reliability for weight calculation [24, 44, 45]. Additionally, the systematic literature review study of Schmidt et al. [22] reported that there was no precise rule for the number of experts involved in the AHP studies, and the method generally did not require large number of experts. The review of 121 AHP studies revealed that the number of experts can be range from 1 to 1300 ($\overline{x}$=109) [22]. Besides, the MAUT method is substantially successful in the assessment of risk preferences, taking uncertainty into account, and changing the formula easily when new attributes or factors are added [12, 44]. Therefore, we believe that the MAUT and the AHP methods suit appropriately to resolve the research question that we focused on in this study.

Another point worth discussing is that the IDCM specialists working in the same institution may tend to make similar decisions with one another. In order to achieve the most accurate result in our decision models, we have been paid a special attention to reach the experts working in different hospitals and institutions. Therefore, the opinions included in the study were collected, as far as possible, from the experts working in different institutions. However, experts who share their views on daily practice in hand hygiene routines, still serve in the same geographical region, and this may be a limitation of our decision models. To overcome such a possible limitation, we plan to develop a web-based application, which aggregates the opinions of the IDCM specialists, as the next step of our research. In this way, we believe we will be able to perform much more comprehensive decision analysis and to understand the attitudes of the IDCM specialists towards hand hygiene more deeply.

## Conclusions

The MAUT and the AHP, decision models developed here indicate that rubbing the hands with ABAS is the most favorable choice for IDCM specialists to prevent nosocomial infection. We believe that our study has the potential to illuminate the key factors underlying IDCM specialists’ behaviors and attitudes towards the hand hygiene. Additionally, employees in IDCM departments should be informed regarding the benefits of ABAS when the optimal hand hygiene preference is decided.

## Additional file

Additional file 1: The Questionnaire utilized in face-to-face interviews to collect expert opinions. (PDF 202 kb)

## Abbreviations

ABAS: Alcohol-based antiseptic solution
AHP: Analytic hierarchy process
CR: Consistency ratio
HCWs: Healthcare workers
IDCM: Infectious diseases and clinical microbiology
MAUT: Multi-attribute utility theory
MCDA: Multi-criteria decision analysis
WHO: World Health Organization

## References

[1] Tartari E, Abbas M, Pires D, de Kraker MEA, Pittet D, World Health Organization SAVE LIVES. Clean your hands global campaign-‘fight antibiotic resistance-it’s in your hands. Clin Microbiol Infect. 2017; 10.1016/j.cmi.2017.04.021 28487167

[2] Mathur P (2011). Hand hygiene: back to the basics of infection control. Indian J Med Res.

[3] Luangasanatip N, Hongsuwan M, Limmathurotsakul D, Lubell Y, Lee AS, Harbarth S (2015). Comparative efficacy of interventions to promote hand hygiene in hospital: systematic review and network meta-analysis. BMJ.

[4] Pittet D, Boyce JM (2001). Hand hygiene and patient care: pursuing the Semmelweis legacy. Lancet Infect Dis.

[5] Boyce JM, Pittet D (2002). Guideline for hand hygiene in health-care settings. Recommendations of the healthcare infection control practices advisory committee and the HIPAC/SHEA/APIC/IDSA hand hygiene task force. Am J Infect Control.

[6] Pittet D, Allegranzi B, Boyce J (2009). The World Health Organization guidelines on hand hygiene in health care and their consensus recommendations. Infect Control Hosp Epidemiol.

[7] Karaaslan A, Kepenekli Kadayifci E, Atıcı S, Sili U, Soysal A, Çulha G (2014). Compliance of healthcare workers with hand hygiene practices in neonatal and pediatric intensive care units: overt observation. Interdiscip Perspect Infect Dis.

[8] Ehrenkranz NJ, Alfonso BC (1991). Failure of bland soap handwash to prevent hand transfer of patient bacteria to urethral catheters. Infect Control Hosp Epidemiol.

[9] Longtin Y, Sax H, Allegranzi B, Schneider F, Pittet D (2011). Videos in clinical medicine. Hand hygiene. N Engl J Med.

[10] Chung S, Kim S, Kim J, Sohn K (2010). Use of multiattribute utility theory for formulary management in a health system. Am J Heal Pharm.

[11] Chang K-Y, Chan K-H, Chang S-H, Yang M-C, Chen TH-H (2008). Decision analysis for epidural labor analgesia with multiattribute utility (MAU) model. Clin J Pain.

[12] Bettinger TL, Shuler G, Jones DR, Wilson JP (2007). Schizophrenia: multi-attribute utility theory approach to selection of atypical antipsychotics. Ann Pharmacother.

[13] Hodder SC, Edwards MJ, Brickley MR, Shepherd JP (1997). Multiattribute utility assessment of outcomes of treatment for head and neck cancer. Br J Cancer.

[14] Giauque WC, Peebles TC (1976). Application of multidimensional utility theory in determining optimal test-treatment strategies for streptococcal sore throat and rheumatic fever. Oper Res.

[15] Carter WB (1992). Psychology and decision making: modelling health behavior with multiattribute utility theory. J Dent Educ.

[16] Carter WB, Beach LR, Inui TS (1986). The flu shot study: using multiattribute utility theory to design a vaccination intervention. Organ Behav Hum Decis Process.

[17] Baker J, McKnew M, Gulledge TR, Ringuest JL (1984). An application of multiattribute utility theory to the planning of emergency medical services. Socio Econ Plan Sci.

[18] Lee I-J, Huang S-Y, Tsou M-Y, Chan K-H, Chang K-Y (2010). Decision analysis for a data collection system of patient-controlled analgesia with a multi-attribute utility model. J Chinese Med Assoc.

[19] Adunlin G, Diaby V, Xiao H (2015). Application of multicriteria decision analysis in health care: a systematic review and bibliometric analysis. Health Expect.

[20] Ashour OM, Okudan Kremer GE (2013). A simulation analysis of the impact of FAHP?MAUT triage algorithm on the emergency department performance measures. Expert Syst Appl.

[21] Wahlster P, Goetghebeur M, Kriza C (2015). Niederl?Nder C, Kolominsky-Rabas P. Balancing costs and benefits at different stages of medical innovation: a systematic review of multi-criteria decision analysis (MCDA). BMC Health Serv Res.

[22] Schmidt K, Aumann I, Hollander I, Damm K, von der Schulenburg J-MG (2015). Applying the analytic hierarchy process in healthcare research: a systematic literature review and evaluation of reporting. BMC Med Inform Decis Mak.

[23] Liberatore M, Nydick R (2008). The analytic hierarchy process in medical and health care decision making: a literature review. Eur J Oper Res.

[24] Suner A, Çelikoğlu CC, Dicle O, Sökmen S (2012). Sequential decision tree using the analytic hierarchy process for decision support in rectal cancer. Artif Intell Med.

[25] Suner A, Karakülah G, Dicle O, Sökmen S, Çelikoğlu CC (2015). CorRECTreatment: a web-based decision support tool for rectal cancer treatment that uses the analytic hierarchy process and decision tree. Appl Clin Inform.

[26] Girou E (2002). Efficacy of handrubbing with alcohol based solution versus standard handwashing with antiseptic soap: randomised clinical trial. BMJ.

[27] Bischoff WE, Reynolds TM, Sessler CN, Edmond MB, Wenzel RP (2000). Handwashing compliance by health care workers: the impact of introducing an accessible, alcohol-based hand antiseptic. Arch Intern Med.

[28] Harris A, Samore M, Nafziger R, DiRosario K, Roghmann M, Carmeli Y (2000). A survey on handwashing practices and opinions of healthcare workers. J. Hosp. Infect..

[29] Naikoba S, Hayward A (2001). The effectiveness of interventions aimed at increasing handwashing in healthcare workers - a systematic review. J Hosp Infect.

[30] Grol R, Grimshaw J (2003). From best evidence to best practice: effective implementation of change in patients’ care. Lancet.

[31] Fishburn PC (1967). Letter to the editor?Additive utilities with incomplete product sets: application to priorities and assignments. Oper Res.

[32] Keeney RL, Raiffa H (1993). Decisions with multiple objectives–preferences and value tradeoffs.

[33] Saaty TL (1977). A scaling method for priorities in hierarchical structures. J Math Psychol.

[34] Saaty TL (2008). Decision making with the analytic hierarchy process. Int J Serv Sci.

[35] Saaty RW (1987). The analytic hierarchy process—what it is and how it is used. Math Model.

[36] Zahedi F (1986). The analytic hierarchy process—a survey of the method and its applications. Interfaces (Providence).

[37] Saaty TL (1990). How to make a decision: the analytic hierarchy process. Eur J Oper Res.

[38] Bhushan N, Rai K. Strategic decision making: applying the analytic hierarchy process: Springer; 2004.

[39] Hair JF, Black WC, Babin BJ, Anderson RE. Multivariate Data Analysis. 6th ed: Pearson Prentice Hall; 2006.

[40] Maury E, Alzieu M, Baudel JL, Haram N, Barbut F, Guidet B (2000). Availability of an alcohol solution can improve hand disinfection compliance in an intensive care unit. Am J Respir Crit Care Med.

[41] Pittet D, Dharan S, Touveneau S, Sauvan V, Perneger TV (1999). Bacterial contamination of the hands of hospital staff during routine patient care. Arch Intern Med.

[42] Zaragoza M, Sallés M, Gomez J, Bayas JM, Trilla A (1999). Handwashing with soap or alcoholic solutions? A randomized clinical trial of its effectiveness. Am J Infect Control.

[43] Huang IB, Keisler J, Linkov I (2011). Multi-criteria decision analysis in environmental sciences: ten years of applications and trends. Sci Total Environ.

[44] Zardari NH, Ahmed K, Shirazi SM, Yusop ZB (2015). Weighting methods and their effects on multi-criteria decision making model outcomes in water resources management.

[45] Kim S-K, Song O (2009). A MAUT approach for selecting a dismantling scenario for the thermal column in KRR-1. Ann Nucl Energy.

