# Supplementary material for: Evaluation of infectious diseases and clinical microbiology specialists’ preferences for hand hygiene: analysis using the multi-attribute utility theory and the analytic hierarchy process methods
Source: BMC Med Inform Decis Mak. 2017 Aug 31;17:129. doi: 10.1186/s12911-017-0528-z (PMC5580304; doi:10.1186/s12911-017-0528-z)
Supplement: Additional file 1: — The Questionnaire utilized in face-to-face interviews to collect expert opinions. (PDF 202 kb) [file 12911_2017_528_MOESM1_ESM.pdf]

Supplementary File 1. The Questionnaire utilized in face-to-face interviews to collect expert opinions

Evaluation of Infectious Diseases and Clinical Microbiology Specialists' Preferences for Hand Hygiene

1. Gender: F □ / M □
2. Age: ____________
3. Institution: ____________
4. Experience year: ____________

This questionnaire is designed to evaluate the choice of infectious diseases and clinical microbiology specialists' preferences for hand hygiene. Please sign the most suitable method below.

**Criteria that affect the preferences for hand hygiene**	**Preferences for Hand Hygiene**									
	**Antimicrobial Soap and Water**					**Alcohol-based antiseptic solution (ABAS)**				
	**1**	**2**	**3**	**4**	**5**	**1**	**2**	**3**	**4**	**5**
**1. Short time application:** Washing the hands with antimicrobial soap and water must be at least 40 to 60 seconds rubbing, with alcohol-based antiseptics must be at least 20 to 40 seconds.										
**2. Glove usage:** Providing hand hygiene before and after glove use..										
**3. Dry and cracked skin:** Side effect of washing with soap and water and/or alcohol-based antiseptic solution due to frequent daily usage.										
**4. Workload of the health workers:** For all health workers such as nurses and allied health care personnel.										
**5. Easy to use:** Easy access to materials, inability to easily reach the sink, not suitable taps for hand washing.										
**6. Intervention type:** Physical contact with patient (handshake, fever measurement, pulse measurement, blood pressure measurement, carrying patient, etc.), physical contact with surrounding environment of the patient (patient beds, linens, tables, chairs, cabinets, etc.), to prevent cross-contamination of different body sites when passing from one process to another in the same patient (urinary catheter, opening intravenous, inserting a nasogastric tube etc.).										
**7. Efficiency:** More effective than other.										

Supplementary File 1. The Questionnaire utilized in face-to-face interviews to collect expert opinions (continues)

I. Among the reasons mentioned in the previous table, please mark the choice is more important for the two options.

II. According to your choice, please mark that how many times your choice is more important than the other option (1-equal importance; 9-extreme importance).

I. Which one is more important, A or B? (Please mark with x)					II. How many times more important? (1-9)								
No	A	x	B	x	Equal importance (1)	(2)	Moderate importance (3)	(4)	Strong importance (5)	(6)	Very strong importance (7)	(8)	Extreme importance (9)
1	Short time application		Glove usage										
2	Short time application		Dry and cracked skin										
3	Short time application		Workload of the staff										
4	Short time application		Easy to use										
5	Short time application		Intervention type										
6	Short time application		Efficiency										
7	Glove usage		Dry and cracked skin										
8	Glove usage		Workload of the staff										
9	Glove usage		Easy to use										
10	Glove usage		Intervention type										
11	Glove usage		Efficiency										
12	Dry and cracked skin		Workload of the staff										
13	Dry and cracked skin		Easy to use										
14	Dry and cracked skin		Intervention type										
15	Dry and cracked skin		Efficiency										
16	Workload of the staff		Easy to use										
17	Workload of the staff		Intervention type										
18	Workload of the staff		Efficiency										
19	Easy to use		Intervention type										
20	Easy to use		Efficiency										
21	Intervention type		Efficiency										

Supplementary File 1. The Questionnaire utilized in face-to-face interviews to collect expert opinions (continues)

I. Among the criteria, please mark the choice is more important for the two hand hygiene options.

II. According to your choice, please mark that how many times your choice is more important than the other option (1-equal importance; 9-extreme importance).

No	Criteria	I. Which one is more important, A or B? (Please mark with x)				II. How many times more important? (1-9)								
		A	x	B	x	Equal importance (1)	(2)	Moderate importance (3)	(4)	Strong importance (5)	(6)	Very strong importance (7)	(8)	Extreme importance (9)
1	Short time application	Antimicrobial Soap and Water		ABAS*										
2	Glove usage	Antimicrobial Soap and Water		ABAS										
3	Dry and cracked skin	Antimicrobial Soap and Water		ABAS										
4	Workload of the staff	Antimicrobial Soap and Water		ABAS										
5	Easy to use	Antimicrobial Soap and Water		ABAS										
6	Intervention type	Antimicrobial Soap and Water		ABAS										
7	Efficiency	Antimicrobial Soap and Water		ABAS										

* Alcohol-based antiseptic solution
